# Neurological and respiratory effects of lung protective ventilation in acute brain injury patients without lung injury: brain vent, a single centre randomized interventional study

**DOI:** 10.1186/s13054-023-04383-z

**Published:** 2023-03-20

**Authors:** Erta Beqiri, Peter Smielewski, Claude Guérin, Marek Czosnyka, Chiara Robba, Lars Bjertnæs, Shirin K. Frisvold

**Affiliations:** 1 Department of Clinical Neurosciences, Neurosurgery Department, University of Cambridge, Cambridge, UK; 2 University of Lyon, Lyon, France; 3 INSERM955, Créteil, France; 4 IRCCS for Oncology and Neuroscience, Policlinico San Martino, Genoa, Italy; 5 Department of Surgical Science Diagnostic and Integrated, University of Genova, Genoa, Italy; 6 Department of Anaesthesia and Intensive Care, University Hospital of North Norway, Tromsø, Norway; 7 Department of Clinical Medicine, UiT the Arctic University of Norway, Tromsø, Norway

**Keywords:** Acute brain injury, Cerebral autoregulation, Intracranial pressure, Lung protective ventilation, Positive end-expiratory pressure, Subarachnoid haemorrhage, Traumatic brain injury, Transpulmonary pressure

## Abstract

**Introduction:**

Lung protective ventilation (LPV) comprising low tidal volume (VT) and high positive end-expiratory pressure (PEEP) may compromise cerebral perfusion in acute brain injury (ABI). In patients with ABI, we investigated whether LPV is associated with increased intracranial pressure (ICP) and/or deranged cerebral autoregulation (CA), brain compensatory reserve and oxygenation.

**Methods:**

In a prospective, crossover study, 30 intubated ABI patients with normal ICP and no lung injury were randomly assigned to receive low VT [6 ml/kg/predicted (pbw)]/at either low (5 cmH_2_O) or high PEEP (12 cmH_2_O). Between each intervention, baseline ventilation (VT 9 ml/kg/pbw and PEEP 5 cmH_2_O) were resumed. The safety limit for interruption of the intervention was ICP above 22 mmHg for more than 5 min. Airway and transpulmonary pressures were continuously monitored to assess respiratory mechanics. We recorded ICP by using external ventricular drainage or a parenchymal probe. CA and brain compensatory reserve were derived from ICP waveform analysis.

**Results:**

We included 27 patients (intracerebral haemorrhage, traumatic brain injury, subarachnoid haemorrhage), of whom 6 reached the safety limit, which required interruption of at least one intervention. For those without intervention interruption, the ICP change from baseline to “low VT/low PEEP” and “low VT/high PEEP” were 2.2 mmHg and 2.3 mmHg, respectively, and considered clinically non-relevant. None of the interventions affected CA or oxygenation significantly. Interrupted events were associated with high baseline ICP (*p* < 0.001), low brain compensatory reserve (*p* < 0.01) and mechanical power (*p* < 0.05).

The transpulmonary driving pressure was 5 ± 2 cmH_2_O in both interventions. Partial arterial pressure of carbon dioxide was kept in the range 34–36 mmHg by adjusting the respiratory rate, hence, changes in carbon dioxide were not associated with the increase in ICP.

**Conclusions:**

The present study found that most patients did not experience any adverse effects of LPV, neither on ICP nor CA. However, in almost a quarter of patients, the ICP rose above the safety limit for interrupting the interventions. Baseline ICP, brain compensatory reserve, and mechanical power can predict a potentially deleterious effect of LPV and can be used to personalize ventilator settings.

*Trial registration* NCT03278769. Registered September 12, 2017.

**Graphical Abstract:**

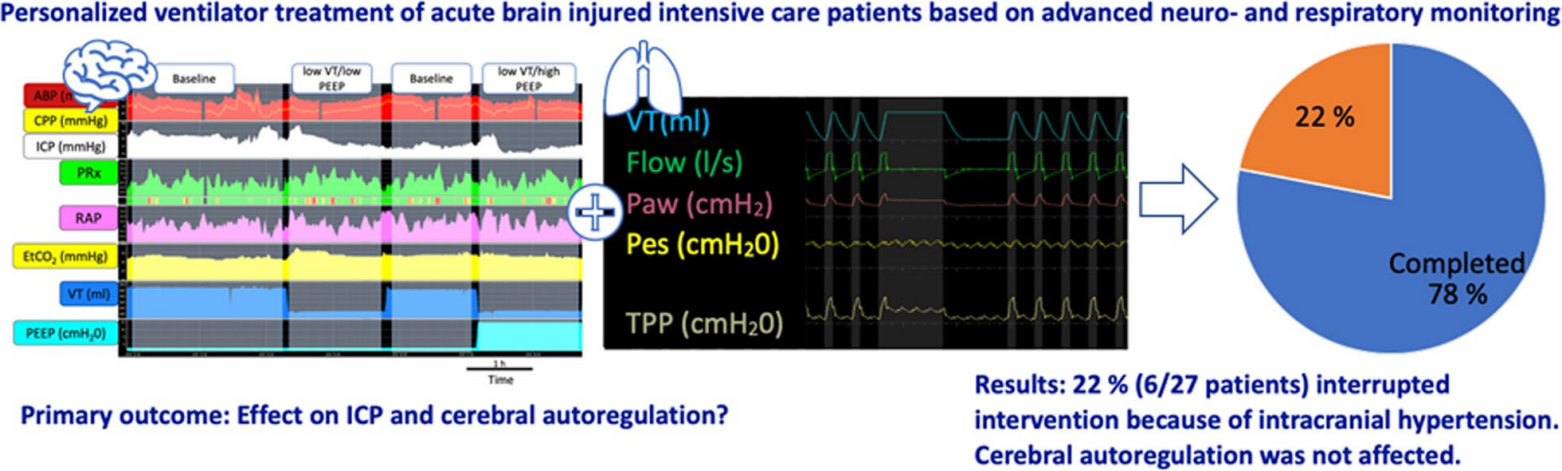

**Supplementary Information:**

The online version contains supplementary material available at 10.1186/s13054-023-04383-z.

## Introduction

The optimal ventilator settings are still debated in patients with severe acute brain injury (ABI) admitted to the intensive care unit (ICU) [1–4]. Lung protective ventilation (LPV) with low tidal volume (VT) and sufficiently high positive end-expiratory pressure (PEEP) to avoid airway collapse is recommended in patients with acute respiratory distress syndrome (ARDS) [5], and may be guided by transpulmonary pressure (TPP) and mechanical power (MP) [6, 7]. Nevertheless, in two recent studies of ICU patients without ARDS, neither the use of different levels of VT nor PEEP, changed patients’ outcome or ventilator-free days [8, 9].

In patients with ABI, lower tidal volumes may improve outcomes [10–12]. A recent expert recommendation on mechanical ventilation (MV) in patients with ABI, suggests using LPV in all patients with ABI and normal ICP, including those without ARDS, but has acknowledged the lack of evidence to support this recommendation [4]. The main concern with application of LPV is the increased intrathoracic pressure, reduced venous return and increased ICP through hypoventilation-induced hypercapnia [13]. The latter changes also have the potential to modify cerebral autoregulation (CA).

It is generally accepted that in patients with ABI, monitoring and treatment of the ICP as the only single cerebral parameter is of limited value [14]. By combining monitoring of cerebral oxygenation, ICP and arterial blood pressure (ABP), the application of waveform analysis, can be used to shed light on the brain pathophysiology in critically ill patient [15]. Two indices are of particular interest from the clinical perspective: the cerebrovascular reactivity index (PRx), and the compensatory reserve index (RAP) [16, 17]. PRx represents a surrogate measure of CA, with a positive index indicating loss of vascular reactivity, consistent with impaired autoregulation [18]. RAP can be described as a surrogate index of global cerebral compliance, with the higher the RAP, the lower the intracranial compliance. Moreover, near-infrared spectroscopy (NIRS) might be used to assess regional cerebral oxygen saturation (rSO_2_) [19].

As a first step, recognizing the need to enrich the evidence behind the recommendations for ventilator management in ABI patients without lung injury, we hypothesized that LPV would not induce significant changes in neuromonitoring-derived variables in this setting, provided that the baseline ICP was less than 22 mmHg. Therefore, the aim was to conduct the brain vent study to investigate the effects of LPV on brain pathophysiology, determined by simultaneous monitoring of TPP, brain oxygenation and indices derived from ICP waveform analysis.

## Materials and methods

### Study design

Brain vent is as a single centre, randomised, crossover interventional clinical trial, which was approved by the Regional Committee for Medical Research Ethics Northern Norway (2017/1941/REK North). The study was conducted between May 2019 and August 2021 in the ICU of a tertiary trauma and neurosurgery centre, affiliated with the University Hospital of North Norway, Tromsø, Norway. The trial is reported according to the Consort guidelines [20].

**Fig. 1:**
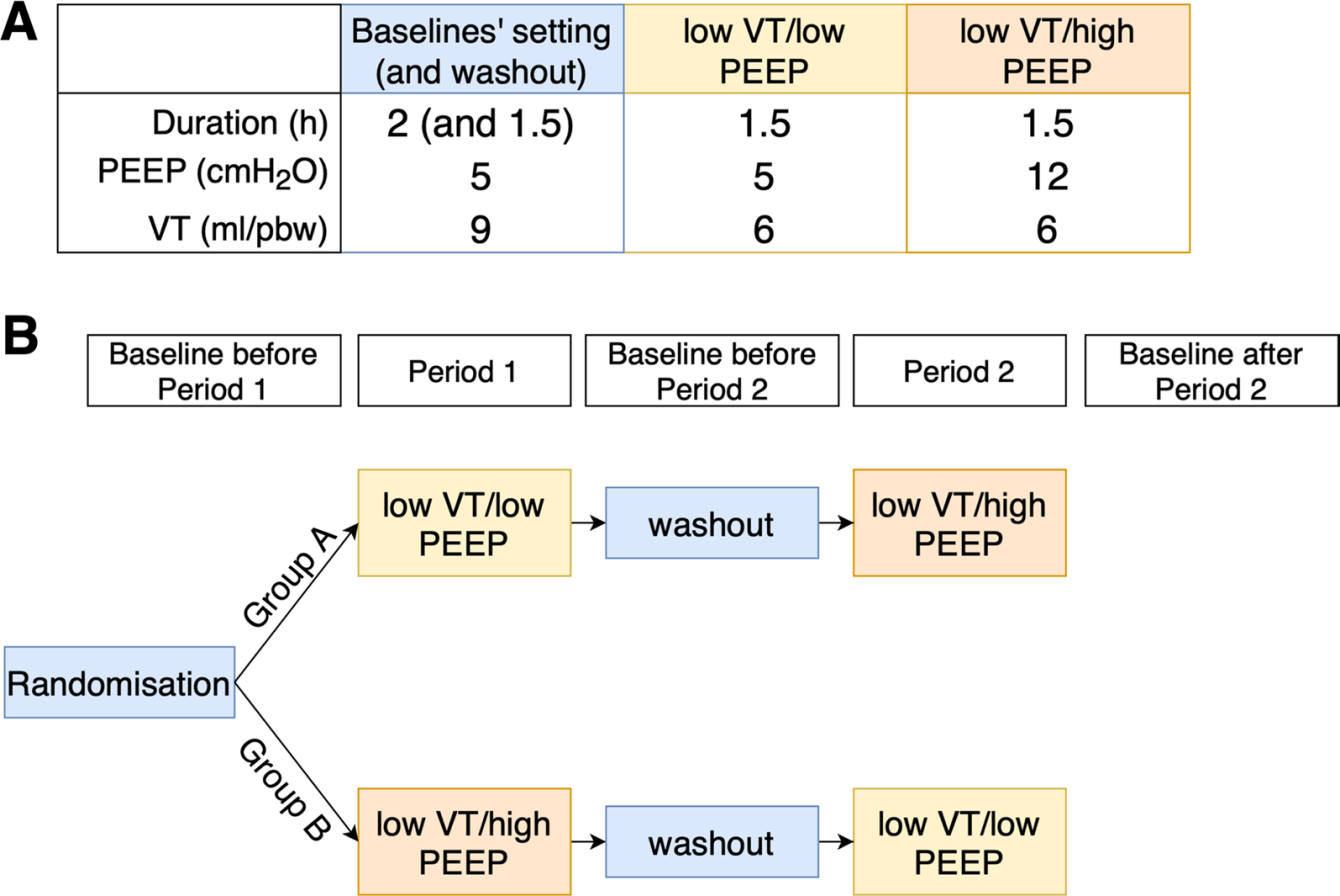
Study design: crossover design and intervention settings. Panel **A** shows the ventilator settings in the different study periods. Panel **B** shows the randomisation scheme and the study periods. During the baselines, patients were ventilated with VT 9 ml/kg/pbw and PEEP 5 cmH_2_0. Group A was first exposed to “low VT/low PEEP” followed by “low VT/high PEEP”. Group B was subjected to the same interventions in reverse order. The length of the periods was chosen to allow PRx to stabilize and thus display less variance. A washout period with baseline ventilator settings was used to reduce the risk for carry-over effect of the intervention. VT, tidal volume; PEEP, positive end expiratory pressure; h, hours; ml/pbw, milliliter per kg predicted body weight

An overview of the study procedures and interventions is presented in Fig. 1. The randomisation was performed through the web-based software program Research Electronic Data Capture (RedCap) [21]. Age (≥ 70, < 70) and type of ABI were used to stratify the two groups. To minimise changes in arterial partial pressure of carbon dioxide (PaCO_2_), minute ventilation was kept as close to the baseline as possible by adjusting the respiratory rate (RR) when VT was changed. Blood gases were sampled after a stabilization period of approximately 30 min after each change in ventilator settings. For safety reasons, the interventions were interrupted if ICP increased above the predefined limit of 22 mmHg for more than 5 min. A healthcare worker blinded to the intervention arm monitored the safety endpoint for the interruption.

### Participants

All consecutive intubated patients on controlled MV with ABI requiring continuous monitoring of intracranial pressure (ICP) were screened for inclusion at the ICU admission. Exclusion criteria were ICP > 22 mmHg, preceding decompressive craniectomy, presence of open external ventricular drainage (EVD), acute hypoxaemic respiratory failure (defined as PaO_2_/FiO_2_ < 300 mmHg and presence of infiltrates on the chest X-ray), a history of severe lung disease, body mass index (BMI) > 35 kg/m^2^, or known cardiac failure. We treated hypovolemia with fluid resuscitation prior to inclusion. All patients were managed according to the local protocols (Additional file 1: Methods 1). The patients were deeply sedated with continuous infusions of propofol and fentanyl to reach a Richmond Agitation Sedation Scale of minus 5 without the use of muscle relaxants. In addition, infusion of midazolam was administrated, if necessary, to reach the sedation target.

### Measurements and data collection

Airway pressure and flow (pneumotachograph) were measured proximal to the endotracheal tube. Oesophageal pressure was measured by using an oesophageal balloon catheter (BA-A-008, MBMed, Argentina). Airway and oesophageal pressures and airflow signals were recorded with FluxView software on a computer connected to a respiratory monitor (FluxMed GrT®, MBMed, Argentina). TPP was calculated as the difference between airway and oesophageal pressures, as described by others [22, 23]. Respiratory mechanics were assessed by using end-inspiratory and end-expiratory holds on the ventilator.

Intensive care monitoring software (ICM + ®, Cambridge Enterprise Ltd, Cambridge UK) [24] was used for continuous recording of high-resolution ICP, ABP, central venous pressure, end-tidal carbon dioxide (EtCO_2_), RR, PEEP, minute ventilation and near-infrared spectroscopy (NIRS)-derived percentage of regional cerebral oxygen saturation (rSO_2_) from both hemispheres (Invos™, Medtronic, USA) [19]. A screenshot of the respiratory monitor and the intensive care monitoring during the data collection is presented in Additional file 1: Fig. S1.

Additionally, the following demographic and clinical data were retrieved from the medical records of the patients: age, sex, BMI, predicted body weight (PBW), Glasgow Coma Scale (GCS), type of brain injury, severity of traumatic brain injury (TBI) by Marshall classification [25], severity of subarachnoid haemorrhage (SAH) by modified Fisher scale [26] and severity of intracerebral haemorrhage (ICH) by intracerebral haemorrhage scale [27], type of ICP device, length of MV, length of ICU stay, ICU mortality, PaCO_2_, pH, arterial partial pressure of oxygen (PaO_2_). The data were collected and managed using RedCap electronic data capture tools [21].

### Outcome measures

The primary outcome was the effect of LPV on ICP and PRx. We identified three endpoints:

1. Number and percentage (%) of patients in whom the ICP response to interventions was below the safety limit of 22 mmHg and therefore could complete the whole protocol.
2. ‘Noninferiority’ of mean ICP, to be understood as an intervention ICP mean value not exceeding the preceding baseline values by more than 3 mmHg. Hence, an increase in ICP ≥ 3 mmHg was considered clinically significant.
3. No worsening in PRx between the intervention value and the preceding baseline value.

The secondary outcome was the effect of LPV on RAP and brain oxygenation, rSO_2_.

### Data analysis

The full set of variables considered in the analysis in addition to outcome variables are presented in the Additional file 1 (Methods 2), together with a detailed data analysis method, including data processing, formulas for calculations of respiratory mechanics and statistical methods. Below, we provide a summary.

### Data processing

ICM + software was used for data preprocessing of the high-resolution recordings prior to statistical analysis. The secondary indices, PRx and RAP were calculated as 5 min window moving Pearson correlation coefficients between 30 consecutive 10-s averages between ABP and ICP, and between intracranial pulse pressure amplitude and ICP, respectively, and updated every minute [28]. Fisher transformation was applied to PRx and RAP prior to further analysis.

Each variable was considered as one average value (for data collected with ICM +) or one single value (for data collected with FluxMed) per each study period and patient.

### Sample size and statistical analysis

The sample size calculation was based on the primary outcome and the crossover study design. Assuming a difference of zero, a standard deviation of 4 mmHg [29] and a noninferiority margin of 3 mmHg, an alpha risk of 0.05, and a beta risk of 0.20 and, a total number of 28 patients were required. We aimed to include 30 patients to count for dropouts.

Continuous variables were tested for normality and are presented as the mean and standard deviation or median and interquartile range, and categorical variables are presented as counts and percentage. Carry-over and group effects were ruled out. The analysis was performed to investigate the treatment effect, assessed as a comparison between the intervention and preceding baseline (paired *t*-test (two-tailed) or Wilcoxon signed-rank test). We assessed the effect of the increase in PEEP by comparing mean values between the two interventions. Noninferiority of mean ICP was tested with paired *t*-test (one-tailed) on mean values (baseline vs intervention).

*Further exploratory analysis*. If the intervention(s) were interrupted, we set out to explore reasons for such outcomes with between-group comparisons. The groups were defined as follows: in the “interrupted group”, the patients had at least one interrupted intervention due to safety reasons; the “completed group” completed the interventions successfully. For between-group comparisons, we used the Mann–Whitney U test and presented the results as boxplots or violin plots. Lastly, we investigated the relationships between the changes induced by the interventions and neurological, haemodynamic, and respiratory variables, using scatterplots and Pearson correlations.

A *p* value < 0.05 was considered statistically significant. Bonferroni corrections for multiple comparisons were performed for the exploratory analysis (between-group comparisons and relationships with changes in ICP). The statistical analysis was performed with R software version 4.0 [30].

## Results

### Baseline characteristics of the patients

**Fig. 2:**
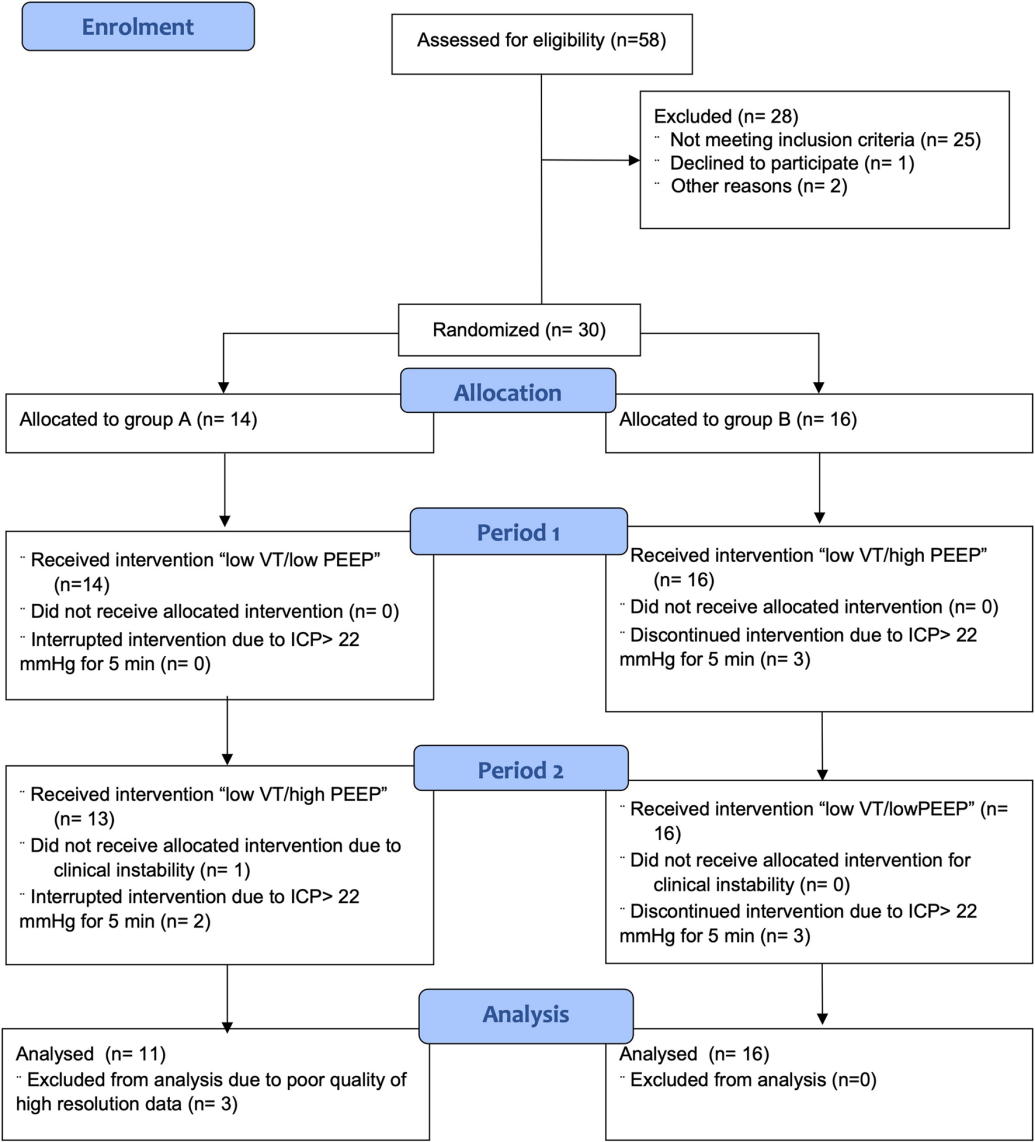
CONSORT flow diagram for crossover study. VT, tidal volume; PEEP, positive end expiratory pressure; ICP, intracranial pressure

Fifty-eight patients were assessed for eligibility, of whom 27 were included in the analysis (Fig. 2). In one patient, the “low VT/high PEEP” intervention was not performed because pneumothorax had developed.

Twenty-three of 27 patients had correct measurements from the oesophageal balloon catheter whereas four installations failed for technical reasons. Patient demographics and clinical characteristics are described in Table 1. Only 4 SAH patients were included, as these patients were commonly treated with open EVD. Therefore, they met more often the exclusion criteria for the study.

**Table 1:** Baseline demographics and clinical characteristics of patients with acute brain injury

Variables	All patients (n = 27)
Male, n (%)	16 (59)
Age (years), Mean ± SD	54 ± 15
BMI (Kg/m^2^), Mean ± SD	26 ± 4
*Type of brain injury*	
Intracerebral haemorrhage, *n* (%)	12 (44)
Subarachnoid haemorrhage, *n* (%)	4 (15)
Traumatic brain Injury, *n* (%)	11 (41)
*Severity scores*	
GCS < 8, *n* (%)	20 (74)
ICH score ^a^, *n* (% of ICH)	
2	1 (8)
3	6 (50)
4	4 (33)
NA	1 (8)
Marshall classification ^b^, (% of TBI)	
II	6 (55)
III	1 (9)
V	4 (36)
Modified Fisher Scale ^c^, *n* (% of SAH)	
I	1 (25)
II	1 (25)
III	1 (25)
IV	1 (25)
*Type of ICP device*	
Parenchymal, *n* (%)	11 (41)
Ventricular + inbuilt sensor, *n* (%)	2 (7)
Ventricular, *n* (%)	14 (52)
*Fluid Balance*	
Fluid balance first day of ICU (ml), Mean ± SD	250 ± 940
Fluid balance whole ICU stay (ml), Mean ± SD	1360 ± 2220
Mechanical Ventilation > 7 days, *n* (%)	14 (52)
ICU stay > 7 days, *n* (%)	16 (59)
Dead in ICU, *n* (%)	6 (22)

^a^The ICH score for risk stratification in intracerebral haemorrhage ranges from 0 to 6, composed of points assigned to the criteria GCS, age, ICH location, ICH volume, and presence of intraventricular blood was assessed in 12 patients

^b^The Marshall classification of traumatic brain injury is based on initial CT scan and ranges from I-VI

^c^The modified Fisher Scale is a method for radiological grading of SAH secondary to intracranial aneurysm rupture. It runs between 1 and 4

*BMI* body mass index; *GCS* Glasgow Coma Scale; *ICH* intracerebral haemorrhage; *TBI* traumatic brain injury; *SAH* subarachnoid haemorrhage; *ICP* intracranial pressure; *ICU* intensive care unit

Baseline respiratory data are displayed in Table 2.

**Table 2:** Baseline characteristics for respiratory variables

Variables	All patients (N = 27)*
PaCO_2_(mmHg)	35 ± 3
PaO_2_/FiO_2_ (mmHg)	343 ± 88
etCO_2_ (mmHg)	30 ± 3
RR (/min)	13 ± 4
Paw_ei_ (cmH_2_O)	14.8 ± 2.5
Pes_ee_ (cmH_2_O)	10.5 ± 4.5
Pes_ei_ (cmH_2_O)	13 ± 4
TPP_ee_ (cmH_2_O)	− 5.3 ± 4.5
TPP_ei_ (cmH_2_O)	1.8 ± 3.6
DP_rs_ (cmH_2_O)	10 ± 3
DP_cw_ (cmH_2_O)	3 ± 2
DP_L,abs_ (cmH_2_O)	7 ± 3
C_rs_ (l/cmH_2_O)	60 (52–73)
C_cw_ (l/cmH_2_O)	269 (143–476)
C_L_ (l/cmH_2_O)	77 (63–131)
R_rs_(cmH_2_O/l/s)	11 (10–14)
Mechanical power (J/min)	9 (6–10)

*N = 23 for variables Pes_ee_, Pes_ei_, TPP_ee_, TPP_ei_, DP_es_, DP_L, abs_, *C*_L_, *C*_cw_, Mechanical Power

Values are presented as mean ± SD or median (1st–3rd quartiles). The baseline settings were VT 9 ml/kg/pbw and PEEP 5 cmH_2_0

*PaCO*_*2*_ arterial partial pressure of carbon dioxide; *PaO*_*2*_ arterial partial pressure of oxygen; *FiO*_*2*_ fraction of inspired oxygen; *PaO*_*2*_*/FiO*_*2*_ P/F ratio; *etCO*_*2*_ end-tidal partial pressure of CO_2_; *RR* respiratory rate; *Paw*_*ei*_ airway pressure end inspiratory; *Pes*_*ee*_ oesophageal pressure end expiratory; *Pes*_*ei*_ oesophageal pressure end inspiratory; *TPP*_*ei*_ transpulmonary pressure end inspiratory, absolute; *TPP*_*ee*_ transpulmonary pressure end expiratory, absolute; *DP*_*rs*_ driving pressure of the respiratory system; *DP*_*es*_ driving pressure of the chestwall; *DP*_*L,abs*_ driving pressure of the lung, absolute value; *C*_*rs*_ compliance respiratory system; *C*_*cw*_ compliance chest wall; *C*_*L*_, compliance lung; *R*_*rs*_, resistance respiratory system

### Effects of LPV on ICP and PRx

**Fig. 3:**
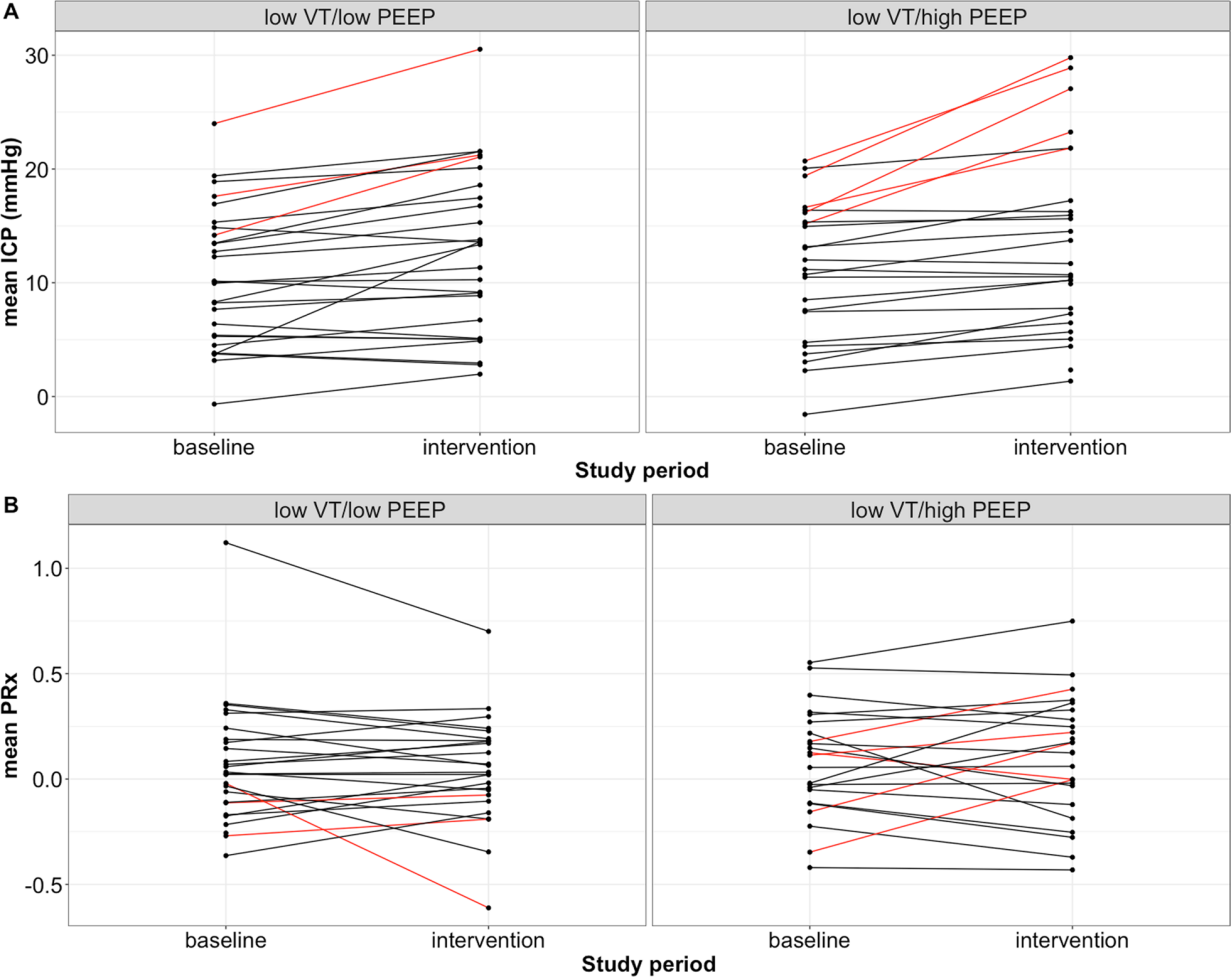
Mean ICP and PRx induced changes. The interrupted interventions were presented in red. The value displayed is the mean value before interruption of the intervention, hence not entirely representative or comparable with not interrupted interventions. Panel (**A**). Mean ICP did not have a clinically important rise as compared with baseline, neither following the “Low VT/low PEEP” intervention nor during “Low VT/high PEEP” (one tailed paired *t*-test for noninferiority, both interventions p = 0.99). Similar results were achieved when including patients in whom the interventions were interrupted. In six of 27 (22%) patients, “Low VT/high PEEP” alone or both interventions were interrupted because the safety limit of ICP > 22 mmHg was reached. In one patient only the “low VT/low PEEP” intervention was interrupted. This patient had an increase in ICP over time, which might explain why the first attempted intervention “low VT/high PEEP” could be tolerated. Panel (**B**). None of the interventions produced significant changes from baseline in mean PRx, neither during intervention “Low VT/low PEEP” (paired *t*-test, p = 0.56) nor during intervention “Low VT/high PEEP” (p = 0.50). Similar results were achieved when including patients in whom the interventions were interrupted. VT, tidal volume; PEEP, positive end expiratory pressure; ICP, intracranial pressure; PRx, pressure reactivity index

In 21 of 27 patients, the interventions with low tidal volume, neither with low PEEP nor with high PEEP caused clinically important increases in ICP (Table 3, Fig. 3). However, eight of 53 (15%) attempted interventions on 6 patients, were interrupted because they reached the prespecified safety limit of ICP > 22 mmHg for more than 5 min (Fig. 2 and 3).

**Table 3:** Effect of LPV on neurological outcome variables

Variables	Low VT/low PEEP			Low VT/high PEEP			*p*^
	Baseline	Intervention	p*	Baseline	Intervention	p*	
ICP (mmHg)	10 ± 6	13 ± 7	< 0.001	11 ± 6	13 ± 8	< 0.001	ns
PRx	0.07 ± 0.3	0.04 ± 0.25	ns	0.08 ± 0.25	0.11 ± 0.28	ns	ns
RAP	0.65 ± 0.51	0.78 ± 0.54	< 0.05	0.62 ± 0.44	0.68 ± 0.46	ns	ns
rSO_2_ left (%)	68 ± 11	67 ± 12	ns	67 ± 11	65 ± 12	ns	< 0.05
rSO_2_ right (%)	68 ± 10	67 ± 10	ns	68 ± 8	65 ± 9	ns	ns

Values are presented as mean ± SD

The results are presented for all patients, independent of whether the intervention was completed or interrupted

p*, p values of paired tests comparing the intervention with the preceding baseline

p^^^, p values of paired tests comparing the two interventions

27 patients were included in the analysis. N for each variable is stated in Additional file 1: Table S1

*ICP* intracranial pressure; *PRx* pressure reactivity index; *RAP* compensatory reserve index; *rSO*_2_ regional cerebral oxygen saturation

The interventions did not produce significant changes in mean PRx in comparison with the baseline values (Table 3, Fig. 3).

Overall, there were no significant differences, neither in mean ICP nor in PRx between the two interventions.

### Effects of LPV on rSO_2 _and RAP

We found no significant differences in the mean rSO_2_ between interventions or in comparison with the preceding baseline (Table 3). Left-sided rSO_2_ displayed a small (2%) difference between the two interventions, which was statistically significant, albeit not considered clinically relevant. RAP tended to increase during the interventions as compared with the preceding baselines but was not different between interventions. Only the increase during the intervention “low VT/low PEEP” was statistically significant (p < 0.05).

### Further exploratory analysis

Additional file 1 (Table S1 and S2) present all the neuromonitoring, haemodynamic and respiratory variables, comparing the study periods, considered in the exploratory analysis.

Below we report the most relevant findings. Additional exploratory analyses on variables associated with the increase in ICP are presented in the Additional file 1 (Fig. S2 and Fig. S3).

*Effect of LPV on respiratory mechanics and gas exchange.* PaCO_2_ was 35 ± 3 mmHg at baseline and 36 ± 3 mmHg in both interventions. Although there was a statistically significant increase in PaCO_2_ between baseline and intervention, the increase was small (< 2 mmHg). TPP_ee_ was higher in the “low VT/high PEEP” intervention. Of note, TPP_ee_ was negative with PEEP 5 cmH_2_O. MP was significantly different both between preceding baselines and interventions and between interventions. The highest MP was in the “low VT/high PEEP” intervention, 13 [11–14] J/min.

**Fig. 4:**
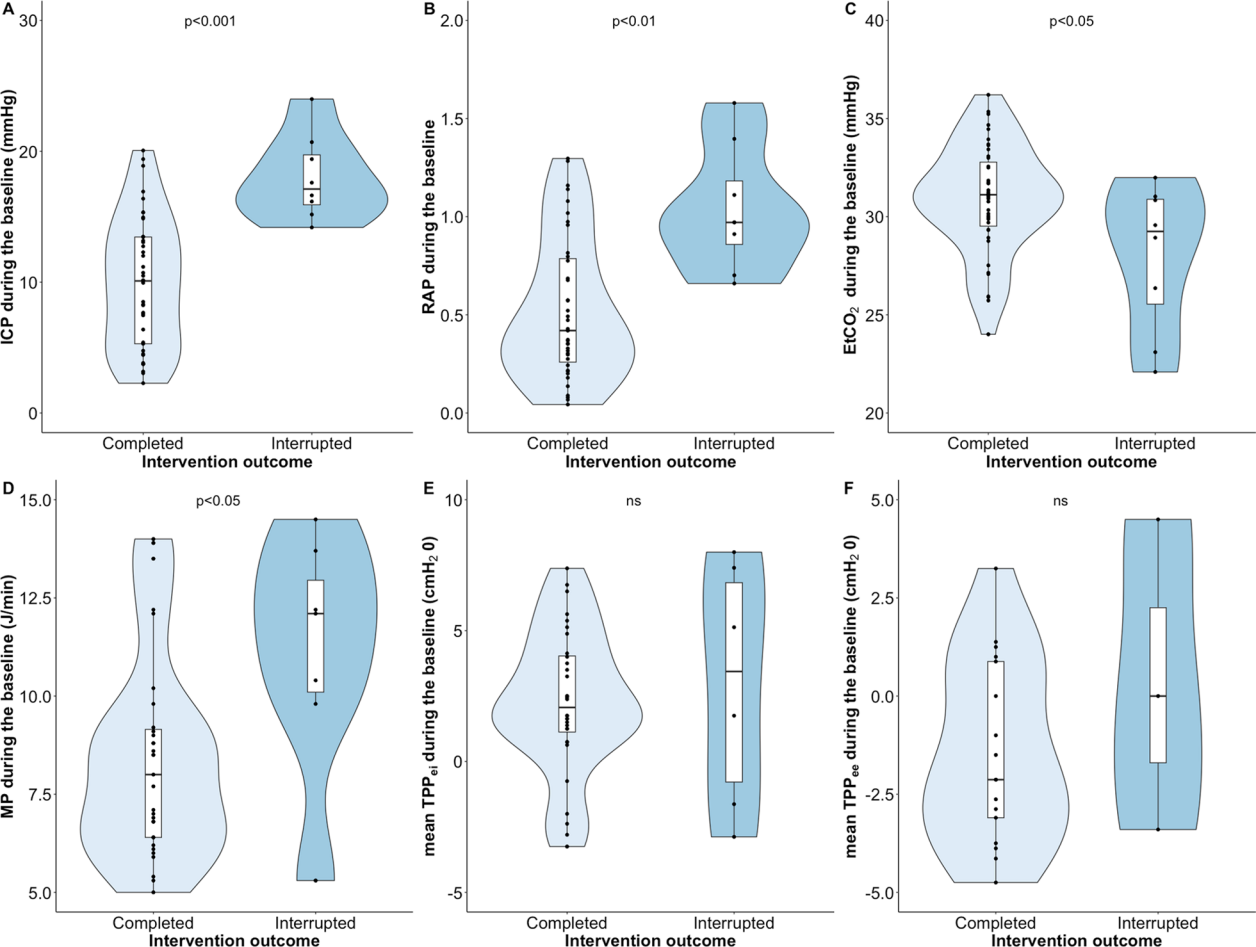
Differences in ICP (**A**), RAP (**B**), EtCO_2_ (**C**), MP (**D**), TPP_ei_ (**E**) and TPP_ee_ (**F**) at baseline preceding interrupted and completed interventions. The data are presented with violin plots and boxplots. A. ICP (n = 8, median ICP = 17.1 (15.9 -19.7) mmHg) at the baseline in the interrupted interventions vs. completed interventions (n = 43, median ICP = 10.0 (4.6–13.3) mmHg). B. RAP at the baseline in the interrupted (n = 8, median RAP = 0.97 (0.86–1.18) vs. the completed (n = 41, median RAP = 0.42 (0.24–0.80) interventions. C. EtCO_2_ at baseline in interrupted (n = 8, median EtCO_2_ = 29.2 (25.5–30.9) mmHg) versus completed (n = 43, median EtCO_2_ = 31.1 (29.5–32.8) mmHg) interventions. D. MP at the baseline in interrupted (n = 7, median MP = 12.1 (10.1–13.0) J/min) vs. completed (n = 33, median MP = 8 (6.4—9.3) J/min) interventions. E and F. Neither TPP_ei_ and TPP_ee_ were significantly different between completed and interrupted events. ICP, intracranial pressure; RAP, compensatory reserve index; EtCO_2_, end-tidal partial pressure of CO_2_; MP, mechanical power; TPP_ei_, transpulmonary pressure end inspiration, absolute; TPP_ee_, transpulmonary pressure end expiration, absolute

*Variables associated with increased ICP that warranted the interruption.* Figure 4 presents differences in physiological variables between the completed and the interrupted group. Baseline ICP, RAP, EtCO_2_ and MP were all significantly different between the completed and the interrupted groups. TPP and compliance of the respiratory system (C_rs_) or lung were not different between completed and interrupted interventions.

Baseline PaCO_2_ was determined to 33.6 ± 1.4 mmHg and 34.9 ± 2.7 mmHg in completed and interrupted interventions, respectively (p = 0.81). The alveolar dead space during the intervention was not significantly different between completed (12 ± 8%) and interrupted (18 ± 9%) interventions (p = 0.19).

## Discussion

In the present clinical, prospective study, the main finding was that most of the patients with ABI had no adverse effects of LPV, neither on ICP nor on PRx, independent of PEEP levels applied in this study. However, nearly one-quarter of the patients exhibited a transient rise in ICP during the intervention, requiring it to be interrupted. The high PEEP strategy was as expected associated with higher TPP_ee_ and MP. To our knowledge, this is the first clinical study attempting to elucidate the effects of lung protective ventilation on brain physiology in ABI patients without lung injury, as assessed by simultaneous monitoring of respiratory mechanics and neurophysiological variables.

Studies dealing with the influence on ICP of various types of MV and associated PEEP levels remain inconclusive [29, 31, 32]. Retrospective post hoc analysis of observational data from patients with ABI without lung injury did not show any significant association between PEEP and ICP [29, 33]. However, such analysis has some limitations; notably, ICP exceeding the predefined target value is treated and therefore cannot become part of a retrospective analysis. In a subgroup of patients with severe lung injury presented in one of the latter studies, the investigators found that there was a 0.3 mmHg increase in ICP for every cmH_2_O rise in PEEP above 5 cmH_2_O [29]. This is consistent with the minor increments in ICP we noticed in patients in whom the interventions were completed.

To date, no prospective phase III clinical trials showing a better outcome with multimodal neuromonitoring have been published [34]. Nevertheless, in recent years there have been attempts to incorporate multimodal monitoring of CA and oxygenation into treatment protocols for ABI [35]. Continuous monitoring of PRx allows for identifying individualised autoregulation-based cerebral perfusion pressure targets in TBI patients. A recent phase II clinical trial has proven the safety and feasibility of this approach, paving the path for outcome trials [36]. However, if different levels of VT and PEEP were to affect CA, independently of PaCO_2_ or CPP, then PRx-based CPP targets would be difficult to implement in clinical practice. In our study, LPV did not affect PRx. Consequently, PRx-based CPP management protocols can most likely be applied regardless of the ventilator settings, provided that PaCO_2_ levels are kept stable.

The modest increase in RAP observed during the intervention periods, suggesting a slightly decrease in cerebral compliance, was consistent with a small and clinically unimportant increment of ICP. rSO_2_ can be considered as an indirect, surrogate measure of cerebral blood flow. The fact that rSO_2_ did not decrease with the application of LPV supports the hypothesis that LPV can be safe in ABI patients. In patients with the combination of ABI and ARDS, Nemer et al. have reported an increase in brain tissue oxygenation during a short trial of high PEEP [37].

### The interaction between neurological and respiratory variables and ICP

In the present study, we specifically targeted tight EtCO_2_ control to minimize the confounding effects of a rise in EtCO_2_ on ICP and PRx. Patients also had stable haemodynamics and hypovolemia was treated prior to the start of the study. This allowed us to look for other mechanisms of intracranial hypertension that made interruption of interventions necessary. Notably, the latter patients experienced a higher ICP level at baseline, and the subsequent rise in ICP might well have emerged in a state of low brain compliance, as determined with the RAP index at the preceding baseline. Our result is supported by an early report that examined the potential role of brain compliance in the ICP response to PEEP, employing cerebral pressure–volume measurements[38]. Contrary to our findings, McGuire and co-workers noticed that patients with increased baseline ICP did not respond with a further rise when exposed to higher PEEP levels [39]. However, brain compliance was not assessed in that study. If ICP waveform analysis is available, we suggest paying special attention to those patients with high RAP.

We observed a tendency towards a wider PaCO_2_–EtCO_2_ gap at the baseline in patients in whom we interrupted the intervention. The present study was not designed to elucidate the interaction between ICP and CO_2_. Therefore, we cannot draw any conclusions from this observation. Monitoring the PaCO_2_–EtCO_2_ gap is easily feasible, and we hope future studies will consider this observation.

MP is described as energy delivered to the respiratory system over time. In neurocritical care patients, increased MP may be related to higher ICU mortality [40]. The present study is the first one to analyse the relationship between MP and cerebral variables in patients with ABI. As expected, MP was highest in the high PEEP intervention. It was also highest at baseline, in patients in whom we interrupted the interventions. Nevertheless, MP was lower than the potential safety limit of MP 17 J/min, indicating that the use of a lower TV and higher RR is acceptable in relation to MP [41]. The pathophysiological mechanisms and the clinical significance of these associations need to be further elucidated and analysed.

In this investigation on non-ARDS patients, we found no relationship between increased TPP and interrupted events. Of note, TPP_ee_ was negative with PEEP 5 cmH_2_O. We cannot exclude the possibility that the patients might be at risk of developing atelectasis by applying PEEP of 5 cmH_2_O or below [42]. Considering the risk for ARDS that the ABI patients are exposed to, further data on TPP from randomised, multi-centre studies are warranted to explore optimal PEEP in patients with ABI, both with and without ARDS.

Our study did not demonstrate significant associations between respiratory compliance and the responses of ICP to LPV. Previous studies of lung compliance and ICP have given contradictory results [31, 43, 44]. Regarding patients with SAH, Chen et al. revealed that chest wall compliance, in contrast to lung or airway compliance, correlated inversely with ICP under exposure to increasing PEEP from 5 cmH_2_O to 15 cmH_2_O [43]. In the latter study, the differences in mean ICP at the two PEEP levels were consistent with those observed in the present study, but with no interrupted events. We admit that their study population was not entirely comparable with ours since both the baseline ICP and the P/F ratios were lower in their study. Investigators of an observational study employing computed tomography, reported a significant increase in ICP following a rise in PEEP from 5 to 15 cmH_2_O [31]. The authors observed that, when applying high PEEP, the increase in ICP correlated inversely with C_rs_. Notably, PaCO_2_ was higher than in our study, and the patient´s head level was positioned flat for computer tomography. These variations can partly explain the different results. Moreover, Mascia et al. reported a significant correlation between ICP, PaCO_2_ and C_rs_ in response to changes in PEEP in patients with moderate to severe lung injury [44]. The cohort consisted of patients on both spontaneous and controlled ventilation.

### Limitations

The present study has some limitations. A major one is that it is a single-centre study with a small sample size. Notably, the interrupted group is too small, to draw any firm conclusions from. The power analysis was based on local retrospective ICP data in parallel with clinical judgement, in combination with a previous observational study [29]. We considered an ICP change of 3 mmHg was considered as clinically significant, in accordance with previous studies [43, 45]. We also thought that the results of this pilot study could inspire a future multicentre trial encompassing more rigid power analysis. Although the numbers used for power analysis should be interpreted with caution, we believe that our study provided solid data, particularly because the patients were thoroughly monitored and treated by a few dedicated physicians and intensive care nurses. All relevant events were documented in real-time during the whole study period. All these precautions most likely contributed to reducing the number and impact of confounding variables.

It is possible, albeit not proven, that another study design with slowly increasing PEEP would have given other changes in ICP. We did not test the response to high tidal volume and high PEEP, because of the risk of overdistention. However, the study design allowed us to explore the effect of low tidal volume and high PEEP separately, which had not been done before. A limitation is also the fact that we did neither have recordings of brain tissue oxygen tension, nor cardiac output measurement, since this type of devices are used sparingly in our ICU in this patient group.

## Conclusion

LPV seems to be safe and feasible for most but not all patients with ABI. In patients with suspected low brain compliance, LPV should be performed under careful monitoring, with an emphasis on respiratory mechanics and ICP waveform-derived variables when available. Baseline ICP, RAP, etCO_2_ and MP were significant predictors of an ICP increase, suggesting that they can be used to individualize ventilator settings in this context. Our findings provide a basis for further clinical investigations of the interaction between ventilator settings and ABI complicated with ARDS, using TPP and multimodal neuromonitoring tools.

## Supplementary Information

**Additional file 1**. Detailed methods, supplemental tables and figures.

## Data Availability

The datasets used and analysed during the current study are available from the corresponding author on reasonable request. Full details of the trial protocol can be found at clinicaltrials.gov/NCT03278769.

## Abbreviations

ABI: Acute brain injury
ABP: Arterial blood pressure
ARDS: Acute respiratory distress syndrome
BMI: Body mass index
C_rs_: Compliance of the respiratory system
CA: Cerebral blood flow autoregulation
CPP: Cerebral perfusion pressure
EVD: External ventricular drainage
EtCO_2_: End-tidal partial pressure of CO_2_
FiO_2_: Fraction of inspired oxygen
ICH: Intracranial haemorrhage
ICM +: Intensive care monitor +
ICP: Intracranial pressure
ICU: Intensive care unit
LPV: Lung protective ventilation
MP: Mechanical power
MV: Mechanical ventilation
NIRS: Near-infrared spectroscopy
PaCO_2_: Arterial carbon dioxide partial pressure
PaO_2_: Arterial oxygen partial pressure
Pbw: Predicted body weight
PEEP: Positive end-expiratory pressure
PRx: Pressure reactivity index
RAP: Compensatory reserve index
RR: Respiratory rate
RedCap: Research electronic data capture
rSO_2_: Regional cerebral oxygen saturation
SAH: Subarachnoid haemorrhage
TBI: Traumatic brain injury
TPP: Transpulmonary pressure
TPP_ei_: End-inspiratory transpulmonary pressure, absolute
TPP_ee_: End-expiratory transpulmonary pressure
VT: Tidal volume
