# Supplementary material for: Neurological and respiratory effects of lung protective ventilation in acute brain injury patients without lung injury: brain vent, a single centre randomized interventional study
Source: Crit Care. 2023 Mar 20;27:115. doi: 10.1186/s13054-023-04383-z (PMC10026451; doi:10.1186/s13054-023-04383-z)
Supplement: Supplementary file 1 — Additional file 1. Detailed methods, supplemental tables and figures. [file 13054_2023_4383_MOESM1_ESM.docx]

**Electronic Supplementary Material**

**Neurological and respiratory effects of lung protective ventilation in acute brain injury patients without lung injury: brain vent, a single centre randomized interventional study**

Authors: Erta Beqiri^1^, Peter Smielewski^1^, Claude Guérin^2,3^, Marek Czosnyka^1^, Chiara Robba^4,5^, Lars Bjertnæs^6,7^, Shirin K Frisvold^6,7^

^1^ Department of Clinical Neurosciences, Neurosurgery Department, University of Cambridge, UK

^2^ University of Lyon, Lyon, France

^3^ INSERM955, Créteil, France

^4^ Policlinico San Martino, IRCCS for Oncology and Neuroscience, Genova, Italy

^5^ Department of Surgical Science Diagnostic and Integrated, University of Genova, Italy

^6^ Department of Anaesthesia and Intensive care, University Hospital of North Norway, Tromsø, Norway

^7^ Department of Clinical Medicine, UiT the Arctic University of Norway, Tromsø, Norway

Corresponding author: Shirin K Frisvold, shirin.frisvold@uit.no

**Table of content Page**

**Additional file 1**

1. **Methods. 1 2**
2. **Methods. 2 2**
3. **Table S 1 7**
4. **Table S 2 10**
5. **Figure S 1 13**
6. **Figure S 2 15**
7. **Figure S 3 16**
8. **List of abbreviations for the additional material 17**

**Additional File 1**

1. **Methods 1- Local clinical protocol for ICU management of ABI patients**

The patients were deeply sedated with continuous intravenous propofol and fentanyl infusion to Richmond agitation scale minus 5. The targets were cerebral perfusion pressure (CPP) of >60 mmHg, partial pressures of arterial carbon dioxide (PaCO_2_) and oxygen (PaO_2_) of 4.3-5 kPa, and over 10 kPa, respectively, and oxygen saturation >96%. CPP was maintained with fluids, vasopressors and/or inotropic agents based on the clinician’s evaluation. The patients were positioned supine with the head of the bed elevated to 20 degrees and ventilated with Servo U ventilators (Maquet, Rastatt, Germany). The ventilator settings throughout the study were: volume controlled mode; inspiratory:expiratory (I:E) ratio 1:2; Inspiratory time 10 %; respiratory rate (RR) as needed to maintain PaCO_2_ within the range of 4.3-5 kPa; fraction of inspired oxygen (FiO_2_) set to maintain an oxygen saturation measured by pulse oximetry of > 96 %; and the airway plateau pressure maintained < 30 cm H_2_O throughout the study.

1. **Methods 2- Data analysis**

Data Processing

ICM+ high resolution data

ICM+ software (ICM+®, Cambridge Enterprise Ltd, Cambridge UK) was used for data pre-processing of high-resolution recordings prior to statistical analysis.

High-resolution data were recorded with ICM+ at 250 Hz. Two researchers (EB and SF) scrutinised visually each recording and assessed the quality of the signals. Three recordings were excluded, one due to technical issues resulting in unacceptable data gaps, two due to unreliable ICP recording associated with frequent external ventricular drainage opening.

The data were first curated manually for large artefacts by an experienced investigator (EB). Further, automated artefact markup was applied. EtCO_2_ values below 20 mmHg or above 70 mmHg were rejected. Arterial blood pressure (ABP) artefacts were removed automatically in case of absence of pulse detection, or when drops in diastolic values to below unphysiological thresholds (15 mmHg) were detected. Automatic intracranial pressure (ICP) artefacts removal used low amplitude values (<0.04 mmHg) to reject artefactual periods which corresponded to either noise or short recording periods during which external ventricular drainage (EVD) was kept open.

Amplitude of ICP (AMP) was calculated as the amplitude of the fundamental harmonic within the frequency range of 40-180 b/min using Fast Fourier Transform.

All recorded signals were down sampled to 0.1 Hz by coarse graining using 10 s, non-overlapping averages. Secondary indices, pressure reactivity index (PRx) and compensatory reserve index (RAP) were calculated as moving 5 min window correlation coefficients between 30 consecutive 10 second averages of ABP and ICP, and AMP and ICP respectively, and updated every minute. PRx and RAP values were Fisher-transformed prior to any statistical analysis to address the <-1;+1> hard constrain of those indices.

A special consideration needs to be made here. In our cohort, sixteen patients had an EVD in place. After the EVD was opened for treating high ICP, the ICP trend would decrease. This decrease in trend is entirely caused by the change in intracranial volume, and hence not related to vasogenic activity, i.e., not driven by slow changes in ABP. Therefore, PRx would wrongly capture (or interpret) the relationship between ABP and ICP as cerebrovascular reactivity. To solve this issue, we applied a digital filter to both ICP and ABP and removed the main trend. As a result, the slow changes created by cerebrospinal fluid volumes changes were removed. We used an infinite response DC-filter with a frequency cut-off of 0.001 Hz.

All variables were further down sampled to 0.0167 Hz by coarse graining using 60 s, non-overlapping averages. Hence, values referred to in this study are average values over 60 seconds. Given this pre-processing, standard deviation of mean ICP and mean ABP were considered as surrogate measure of the power of the slow wave components in ICP (ICP_sw_) and ABP (ABP_sw_).

For each patient, we exported descriptive statistics values of each variable for each study period, using the event tool implementation in ICM+. Mean values were considered for all variables for further analysis.

Respiratory data

Data from the respiratory monitor (FluxMed GrT®, MBMed, Argentina) were extracted as one value per period at the end-inspiratory and end-expiratory hold respectively.

1. The following variables were retrieved as absolute values from Fluxmed:

End-inspiratory (Paw_ei_) and end-expiratory airway pressure (PEEP), end-inspiratory and end-expiratory oesophageal pressures (Pes_ei_ and Pes_ee_ respectively), and end-inspiratory and end-expiratory transpulmonary pressure (TPP_ei_ and TPP_ee_ respectively).

1. Compliance of the respiratory system (C_rs_) and resistance of the respiratory system (R_rs_) of the respiratory system, were retrieved from the ventilator during inspiratory and expiratory holds.
2. The differential pressures were calculated during inspiratory and expiratory hold with the absolute method as follows:
   1. Lung compliance (C_L)_ and chest wall compliance (C_cw_) were calculated as C_L_ = VT/DP_L, abs_ and C_cw_ = VT/DP_cw_ respectively, where VT is tidal volume, DP_L, abs_ is the absolute value of the driving pressure of the lung, and DP_cw_ is driving pressure of the chest wall.
   2. Driving pressure (DP) of the respiratory system as DP_rs_= Paw_ei_ – PEEP; DP of the lung, absolute (DP_L, abs_) = TPP_ei_ – TPP_ee_; DP_cw_= Pes_ei_ - Pes_ee_.
3. The alveolar dead space (V_Dalv_) was calculated as (PaCO_2_ -EtCO_2_)/PaCO_2_

Predicted body weight (pbw) was calculated by the ventilator to height (in centimetres – 100 for male and height (in centimetres) – 105 for women.

Mechanical power (MP) of the respiratory system in J/min was calculated with the formula: MP= RR * (∆VT^2^ * (1 /2 * El_rs_ + RR * (1 + I : E)/60 * I : E * R_rs_) + ∆VT * PEEP) where VT is the tidal volume in liters, El_rs_ is the elastance of the respiratory system, I:E is the inspiratory-to-expiratory time ratio, and R_rs_ is the airway resistance.

Statistical analysis

The full set of physiological variables considered in the analysis was as follows:

1. Cerebral and haemodynamic variables: RAP, AMP, regional cerebral oxygen saturation (rSO_2_), power of slow waves in ICP (ICP_sw_) and ABP (ABP_sw_), ABP, CPP, central venous pressure (CVP).
2. Gas exchange and respiratory mechanics variables: PaCO_2_, PaO_2_, pH, PaO_2_/FiO_2_, EtCO_2_, RR, Paw_ei_, TPP_ei_, TPP_ee_, DP_rs_, DP_L, abs_, DP_cw_, C_rs_, C_cw_, C_L_, R_rs_, MP.

Statistical analysis was performed with R statistical version 4.0. Normality of continuous variables was assessed with histograms and Shapiro-Wilks test. Homogeneity of variance was assessed. Continuous variables were presented as mean and standard deviation (SD) or median and interquartile range (IQR), and categorical variables (for demographic data) as counts (%). Outliers were evaluated.

Statistical analysis strategy

The assumptions of the cross-over design (absence of carry-over effect and absence of group (A vs B) effect) were tested with linear mixed effect models using type of intervention (low VT/low PEEP, low VT/high PEEP), group (A and B) and period of intervention (first or second) as a fixed effect, with interaction terms, and the patient as the random effect variable. Furthermore, the absence of carry-over effect was tested with repeated measurements ANOVA test on baseline or washout periods for each variable. These investigations showed that TPP_ei_, DP_es_, C_cw_ had a group effect and DP_L_ and C_L_ had a carry-over effect. Hence the effect of high PEEP (as opposed to low PEEP) on these variables should not be considered.

The treatment effects were evaluated with differences in values between the intervention and the baseline period preceding each intervention (see primary and secondary objective). For this purpose, we used parametric or non-parametric paired tests. Furthermore, we assessed the effect of the increase in PEEP by comparing mean values of physiological variables between the two interventions (parametric or non-parametric paired tests).

Primary outcome

We counted number of interventions that were interrupted and reported in terms of number of patients in whom at least one interruption had occurred.

We performed paired two-tailed t-test on average values (baseline vs intervention) for PRx and non-inferiority test with one-tailed paired t-test on average values (baseline vs intervention) for ICP. In these calculations the intervention values were considered as mean ICP – 3 mmHg.

Secondary outcome

We assessed the treatment effect on rSO_2_ and RAP (paired t-test).

Further exploratory analysis

If the intervention(s) were interrupted, we set out to explore reasons for such outcome, by looking at cerebral, haemodynamic, and respiratory variables (listed above) in the period preceding the intervention.

We performed between-groups comparisons with Mann-U tests and visualised with box-plots and violin-plots. The groups were defined as follows: “interrupted group” comprising of patients in whom at least one intervention had been interrupted for safety reasons; in the “completed group” the patients had completed the study intervention successfully. Bonferroni correction for multiple comparisons was assessed (n=30). In the same manner, we examined differences in physiological variables between interrupted group and the completed group in the intervention period (n=30 for multiple comparisons).

In an attempt to explore the reasons for the rise in ICP, or the change in PRx, if appropriate, we investigated the relationships between the changes in mean values of ICP and/or PRx induced by the intervention, and the changes in variables listed above as ‘full set of physiological variables considered in the analysis’. Changes induced by the intervention were considered as the difference in mean values between the intervention and preceding baseline. We used scatterplots and Pearson correlation coefficients. Bonferroni correction for multiple comparisons was assessed (n=29). In the same fashion we looked at the relationship between changes in ICP and baseline values (n=30 for multiple comparisons).

The effect of the increase in PEEP on ICP was assessed by comparing mean ICP values between the two interventions (paired t-test). The effect of change in VT on ICP was assessed by comparing mean ICP values from the “low VT/low PEEP” period and the preceding baseline (paired t-test).

Lastly, we looked at the treatment effect for both interventions and at the effect of PEEP on each physiological variable (paired t-test or Wilcoxon-test).

Significance level for all tests was set at 0.05. Bonferroni correction for multiple comparisons was performed for the exploratory analysis, as described above. However, given the exploratory nature of this analysis, we only reported the non-corrected p-values.

1. **Table S 1- Comparison of main variables**

| **Variable** | **low VT/low PEEP** | | | | | **low VT/high PEEP** | | | | |  |
| --- | --- | --- | --- | --- | --- | --- | --- | --- | --- | --- | --- |
|  | **baseline** | | **intervention** | |  | **baseline** | | **intervention** | |  | |
|  | **N** | **Mean (SD) or Median (IQR)** | **N** | **Mean (SD) or Median (IQR)** | **p*** | **N** | **Mean (SD) or Median (IQR)** | **N** | **Mean (SD) or Median (IQR)** | **p*** | **p^** |
| **Cerebral and haemodynamics variables** | | | | | | | | | | | |
| ICP (mmHg) | 27 | 10 ± 6 | 27 | 13 ± 7 | <0.001 | 24 | 11 ± 6 | 26 | 13 ± 8 | <0.001 | ns |
| ICP_sw_ (mmHg) | 27 | 1.18 (0.76–1.71) | 27 | 1.18 (0.64–1.57) | ns | 24 | 1.05 (0.52–1.92) | 26 | 1.09 (0.48–1.87) | ns | ns |
| CPP (mmHg) | 27 | 68 ± 7 | 27 | 65 ± 6 | <0.001 | 24 | 67 ± 4 | 26 | 63 ± 8 | <0.001 | ns |
| AMP (mmHg) | 27 | 2 ± 2 | 27 | 3 ± 2 | <0.001 | 24 | 2 ± 2 | 26 | 2 ± 2 | ns | ns |
| PRx | 26 | 0.07 ± 0.3 | 25 | 0.04 ± 0.25 | ns | 23 | 0.08 ± 0.25 | 25 | 0.11 ± 0.28 | ns | ns |
| RAP | 26 | 0.65 ± 0.51 | 25 | 0.78 ± 0.54 | <0.05 | 23 | 0.62 ± 0.44 | 25 | 0.68 ± 0.46 | ns | ns |
| rSO_2_ left (%) | 18 | 68 ± 11 | 19 | 67 ± 12 | ns | 14 | 67 ± 11 | 17 | 65 ± 12 | ns | <0.05 |
| rSO_2_ right (%) | 19 | 68 ± 10 | 20 | 67 ± 10 | ns | 16 | 68 ± 8 | 18 | 65 ± 9 | ns | ns |
| ABP (mmHg) | 27 | 79 ± 8 | 27 | 78 ± 8 | ns | 24 | 78 ± 7 | 26 | 76 ± 7 | <0.05 | ns |
| ABP_sw_ (mmHg) | 26 | 3.1 (2.2–4.37) | 26 | 2.75 (1.83–4.11) | ns | 23 | 2.66 (2.17–4.08) | 25 | 3.21 (2.31–4.15) | ns | ns |
| CVP | 25 | 6 ± 4 | 26 | 5 ± 3 | ns | 23 | 5 ± 3 | 25 | 7 ± 4 | <0.05 | ns |
| **Gas exchange and respiratory mechanics variables** | | | | | | | | | | | |
| PaCO_2_(mmHg) | 27 | 34.8 ± 2.6 | 25 | 36.3 ± 2.9 | <0.05 | 24 | 34.5 ± 2.6 | 25 | 36.2 ± 3 | <0.05 | ns |
| PaO_2_ (mmHg) | 27 | 103 (94–116) | 26 | 104 (89–115) | ns | 24 | 105 (98–120) | 24 | 101 (95–106) | ns | ns |
| PaO_2_/FiO_2_ (mmHg) | 26 | 340 ± 94 | 26 | 338 ± 98 | ns | 24 | 341 ± 90 | 24 | 330 ± 82 | ns | ns |
| pH | 13 | 7.43 ± 0.05 | 26 | 7.43 ± 0.05 | ns | 12 | 7.42 ± 0.05 | 21 | 7.44 ± 0.05 | ns | ns |
| etCO2 (mmHg) | 27 | 31 ± 3 | 27 | 31 ± 3 | <0.001 | 24 | 30 ± 3 | 26 | 31 ± 3 | <0.05 | ns |
| RR (/min) | 27 | 13 ± 3 | 27 | 19 ± 4 | <0.001 | 24 | 14 ± 3 | 26 | 19 ± 3 | <0.001 | ns |
| VT/PBW (ml/Kg) | 27 | 9 (8–9) | 27 | 6 (6–6) | <0.001 | 24 | 9 (8–9) | 26 | 6 (6–6) | <0.001 | ns |
| V_Dalv_ | 27 | 12 ± 10 | 25 | 13 ± 9 | <0.05 | 24 | 12 ± 8 | 25 | 13 ± 9 | ns | ns |
| PEEP (cmH_2_0) | 27 | 5 (5–5) | 27 | 5 (5–5) | ns | 24 | 5 (5–5) | 26 | 12 (12–12) | <0.001 | <0.001 |
| Paw_ei_ (cmH_2_0) | 21 | 14.3 ± 2.3 | 21 | 12.4 ± 1.6 | <0.001 | 19 | 15.2 ± 2.4 | 21 | 20 ± 1.7 | <0.001 | (-) |
| Pes_ee_ (cmH_2_0) | 21 | 9.7 ± 4.2 | 21 | 9.4 ± 4.0 | ns | 19 | 10.2 ± 5.0 | 21 | 11.0 ± 3.6 | ns | ns |
| Pes_ei_ (cmH_2_0) | 21 | 12.5 ± 4.0 | 21 | 11.12 ± 4.2 | <0.05 | 19 | 13.1 ± 4.5 | 21 | 13.4 ± 3.9 | ns | ns |
| TPP_ee_ (cmH_2_0) | 21 | -4.4 ± 4.1 | 21 | -4.1 ± 4.4 | ns | 19 | -5.2 ± 4.9 | 21 | 1.4 ± 3.5 | <0.001 | <0.001 |
| TPP_ei_ (cmH_2_0) | 21 | 1.9 ± 3.1 | 21 | 1.3 ± 3.6 | <0.05 | 19 | 2.1 ± 3.8 | 21 | 6.8 ± 3.7 | <0.001 | (-) |
| DP_rs_ (cmH_2_0) | 21 | 9 ± 2 | 21 | 7 ± 2 | <0.001 | 19 | 10 ± 2 | 21 | 8 ± 2 | <0.001 | (-) |
| DP_es_ (cmH_2_0) | 21 | 3 ± 2 | 21 | 2 ± 1 | ns | 19 | 3 ± 2 | 21 | 2 ± 1 | ns | (-) |
| DP_L_ (cmH_2_0) | 21 | 6 ± 3 | 21 | 5 ± 2 | <0.05 | 19 | 7 ± 3 | 21 | 5 ± 2 | <0.05 | ns |
| C_rs_ (ml/cmH_2_0) | 27 | 63 (54–77) | 26 | 55 (48–68) | <0.001 | 24 | 62 (56–70) | 24 | 54 (44–58) | <0.05 | ns |
| C_L_ (ml/cmH_2_0) | 21 | 86 (68–138) | 21 | 71 (59–100) | ns | 19 | 77 (62–121) | 21 | 68 (63–103) | ns | ns |
| C_cw_ (ml/cmH_2_0) | 21 | 245 (124–463) | 21 | 252 (159–425) | ns | 19 | 222 (143–297) | 21 | 173 (116–317) | ns | (-) |
| R_rs_(cmH_2_0/l/s) | 27 | 11 (10–14) | 26 | 11 (9–13) | ns | 24 | 11 (10–13) | 24 | 11 (10–13) | ns | ns |
| Mechanical power (J/min) | 21 | 8 (6–10) | 20 | 7 (6–10) | <0.05 | 19 | 9 (7–11) | 20 | 13 (11–14) | <0.001 | <0.001 |

The descriptive results are presented for all patients, independently of the outcome of the intervention (completed or interrupted).

p*, p values of paired tests comparing the intervention with the preceding baseline.

p^^^, p values of paired tests comparing the two interventions.

(-), the two interventions could not be compared because of group or carry-over effect.

The intervention “low VT/low PEEP” generated a significant reduction of TPP_ei_ in comparison with baseline (2 ± 2.9 vs 1.2 ± 3.3 respectively, p<0.05). Of note TPP_ei_ had a significant group effect. The intervention “low VT/high PEEP” was associated with an increase in TPP_ee,_ as compared with baseline (-5 ± 4.2 vs 1.4 ± 3.4 respectively, p< 0.001). The increase in PEEP produced an increase in CVP.

ICP, intracranial pressure; CPP, cerebral perfusion pressure; AMP, amplitude of ICP; PRx, pressure reactivity index; RAP, compensatory reserve index; rSO_2_, regional cerebral oxygen saturation; ABP, arterial blood pressure; CVP, central venous pressure; PaCO_2_, arterial partial pressure of carbon dioxide; PaO_2_, Arterial partial pressure of oxygen; FiO_2_, fraction of inspired oxygen; EtCO_2_, end-tidal partial pressure of CO_2_; RR, respiratory rate; Paw_ei_, end-inspiratory airway pressure;

Pes_ee_, end-expiratory oesophageal pressure; Pes_ei_, end-inspiratory oesophageal pressure; TPP_ei_, end-inspiratory transpulmonary pressure, absolute; TPP_ee_, end-expiratory transpulmonary pressure, absolute; DP_rs_, driving pressure of the respiratory system; DP_L,abs_, driving pressure of the lung, absolute value; C_rs_, compliance of the respiratory system; C_L_, compliance of the lung; R_rs_, resistance of the respiratory system.

1. **Table S 2- Comparison of main variables, for patients that completed theinterventions**

| **Variable** | **low VT/low PEEP** | | | | | **low VT/high PEEP** | | | | |  |
| --- | --- | --- | --- | --- | --- | --- | --- | --- | --- | --- | --- |
|  | **baseline** | | **intervention** | |  | **baseline** | | **intervention** | |  | |
|  | **N** | **Mean (SD) or Median (IQR)** | **N** | **Mean (SD) or Median (IQR)** | **p*** | **N** | **Mean (SD) or Median (IQR)** | **N** | **Mean (SD) or Median (IQR)** | **p*** | **p^** |
| **Cerebral and haemodynamics variables** | | | | | | | | | | | |
| ICP (mmHg) | 24 | 9 ± 5 | 24 | 11 ± 6 | <0.05 | 19 | 9 ± 6 | 21 | 10 ± 5 | <0.001 | ns |
| ICP_sw_ (mmHg) | 24 | 1.11 (0.71–1.63) | 24 | 1.04 (0.51–1.37) | ns | 19 | 1.02 (0.51–1.95) | 21 | 0.93 (0.47–1.29) | ns | ns |
| CPP (mmHg) | 24 | 69 ± 7 | 24 | 65 ± 6 | <0.05 | 19 | 67 ± 5 | 21 | 65 ± 7 | <0.001 | ns |
| AMP (mmHg) | 24 | 2 ± 1 | 24 | 2 ± 2 | <0.05 | 19 | 2 ± 1 | 21 | 2 ± 1 | ns | <0.05 |
| PRx | 23 | 0.09 ± 0.3 | 22 | 0.09 ± 0.22 | ns | 18 | 0.11 ± 0.26 | 20 | 0.09 ± 0.31 | ns | ns |
| RAP | 23 | 0.61 ± 0.53 | 22 | 0.74 ± 0.56 | <0.001 | 18 | 0.5 ± 0.37 | 20 | 0.52 ± 0.31 | ns | ns |
| rSO_2_ left (%) | 16 | 69 ± 12 | 17 | 68 ± 12 | ns | 14 | 67 ± 11 | 16 | 66 ± 12 | ns | <0.05 |
| rSO_2_ right (%) | 17 | 69 ± 9 | 18 | 68 ± 9 | ns | 14 | 68 ± 8 | 16 | 65 ± 10 | ns | ns |
| ABP (mmHg) | 24 | 78 ± 8 | 24 | 77 ± 7 | ns | 19 | 77 ± 7 | 21 | 76 ± 8 | <0.05 | ns |
| ABP_sw_ (mmHg) | 23 | 3.16 (2.22–4.62) | 23 | 3.07 (1.84–4.43) | ns | 18 | 3.28 (2.65–4.62) | 20 | 3.32 (2.47–4.15) | ns | ns |
| CVP | 23 | 6 ± 4 | 24 | 5 ± 3 | ns | 19 | 5 ± 3 | 21 | 7 ± 4 | <0.05 | <0.05 |
| **Gas exchange and respiratory mechanics variables** | | | | | | | | | | | |
| PaCO_2_ (mmHg) | 24 | 35 ± 2.7 | 24 | 36.2 ± 3 | <0.05 | 19 | 34.8 ± 2.8 | 21 | 36.4 ± 3.2 | <0.05 | ns |
| PaO_2_ (mmHg) | 24 | 101 (94–121) | 24 | 104 (88–117) | <0.05 | 19 | 104 (98–113) | 20 | 101 (96–106) | ns | ns |
| PaO2/FiO2 (mmHg) | 23 | 350 ± 96 | 24 | 343 ± 101 | ns | 19 | 345 ± 96 | 20 | 332 ± 86 | ns | ns |
| pH | 12 | 7.43 ± 0.05 | 23 | 7.43 ± 0.05 | ns | 11 | 7.42 ± 0.04 | 17 | 7.43 ± 0.05 | ns | ns |
| etCO_2_ (mmHg) | 24 | 31 ± 3 | 24 | 32 ± 3 | <0.05 | 19 | 31 ± 3 | 21 | 32 ± 3 | <0.05 | ns |
| RR (/min) | 24 | 13 ± 3 | 24 | 18 ± 4 | <0.001 | 19 | 13 ± 2 | 21 | 18 ± 3 | <0.001 | ns |
| VT/PBW (ml/Kg) | 24 | 9 (8–9) | 24 | 6 (6–6) | <0.001 | 19 | 9 (8–9) | 21 | 6 (6–6) | <0.001 | ns |
| PEEP (cmH20) | 24 | 5 (5–5) | 24 | 5 (5–5) | ns | 19 | 5 (5–5) | 21 | 12 (12–12) | <0.001 | <0.001 |
| Paw_ei_ (cmH20) | 19 | 14.1 ± 2.4 | 18 | 12.1 ± 1.6 | <0.001 | 14 | 15.4 ± 2.3 | 17 | 20.4 ± 1.6 | <0.001 | (-) |
| Pes_ee_ (cmH_2_0) | 19 | 9.5 ± 3.6 | 18 | 9.1 ± 3.8 | ns | 14 | 10.2 ± 5.0 | 17 | 10.1 ± 4.1 | ns | ns |
| Pes_ei_ (cmH_2_0) | 19 | 12.3 ± 3.9 | 18 | 10.9 ± 3.9 | <0.05 | 14 | 13.4 ± 3.8 | 17 | 13.4 ± 3.6 | ns | ns |
| TPP_ee_ (cmH20) | 19 | -4.2 ± 3.8 | 18 | -3.9 ± 3.9 | ns | 14 | -5 ± 4.2 | 17 | 1.4 ± 3.4 | <0.001 | <0.001 |
| TPP_ei_ (cmH20) | 19 | 2 ± 2.9 | 18 | 1.2 ± 3.3 | <0.05 | 14 | 2.1 ± 3.2 | 17 | 7 ± 3.5 | <0.001 | (-) |
| DP_rs_ (cmH20) | 19 | 9 ± 2 | 18 | 7 ± 2 | <0.001 | 14 | 10 ± 2 | 17 | 8 ± 2 | <0.001 | (-) |
| DP_es_ (cmH20) | 19 | 3 ± 2 | 18 | 2 ± 1 | <0.05 | 14 | 3 ± 2 | 17 | 2 ± 1 | ns | (-) |
| DP_L_ (cmH20) | 19 | 6 ± 3 | 18 | 5 ± 2 | ns | 14 | 7 ± 3 | 17 | 6 ± 2 | <0.05 | ns |
| C_rs_ (ml/cmH20) | 24 | 63 (58–77) | 24 | 56 (50–70) | <0.001 | 19 | 59 (55–70) | 20 | 54 (43–57) | ns | ns |
| C_L_ (ml/cmH20) | 19 | 86 (66–139) | 18 | 84 (61–108) | ns | 14 | 75 (62–128) | 17 | 67 (63–103) | ns | ns |
| C_cw_ (ml/cmH20 | 19 | 245 (124–460) | 18 | 281 (159–445) | ns | 14 | 222 (133–271) | 17 | 173 (113–283) | ns | (-) |
| R_rs_ (cmH20/l/s) | 24 | 11 (10–12) | 24 | 11 (9–13) | ns | 19 | 11 (10–14) | 20 | 11 (9–13) | ns | ns |
| Mechanical power (J/min) | 19 | 8 (6–9) | 18 | 6.4 (5.4–8.3) | <0.05 | 14 | 9 (7–9) | 16 | 12 (11–14) | <0.001 | <0.001 |

ICP, intracranial pressure; CPP, cerebral perfusion pressure; AMP, amplitude of ICP; PRx, pressure reactivity index; RAP, compensatory reserve index; rSO_2_, regional cerebral oxygen saturation; ABP, arterial blood pressure; CVP, central venous pressure; PaCO_2_, arterial partial pressure of carbon dioxide; PaO_2_, Arterial partial pressure of oxygen; FiO_2_, fraction of inspired oxygen; EtCO_2_, end-tidal partial pressure of CO_2_; RR, respiratory rate; Paw_ei_, end-inspiratory airway pressure;

Pes_ee_, end-expiratory oesophageal pressure; Pes_ei_, end-inspiratory oesophageal pressure; TPP_ei_, end-inspiratory transpulmonary pressure, absolute; TPP_ee_, end-expiratory transpulmonary pressure, absolute; DP_rs_, driving pressure of the respiratory system; DP_L,abs_, driving pressure of the lung, absolute value; C_rs_, compliance of the respiratory system; C_L_, compliance of the lung; R_rs_, resistance of the respiratory system.

1. **Fig. S 1- Screenshot displaying multimodality monitoring variables of a patient enrolled in BrainVent.**

A

**
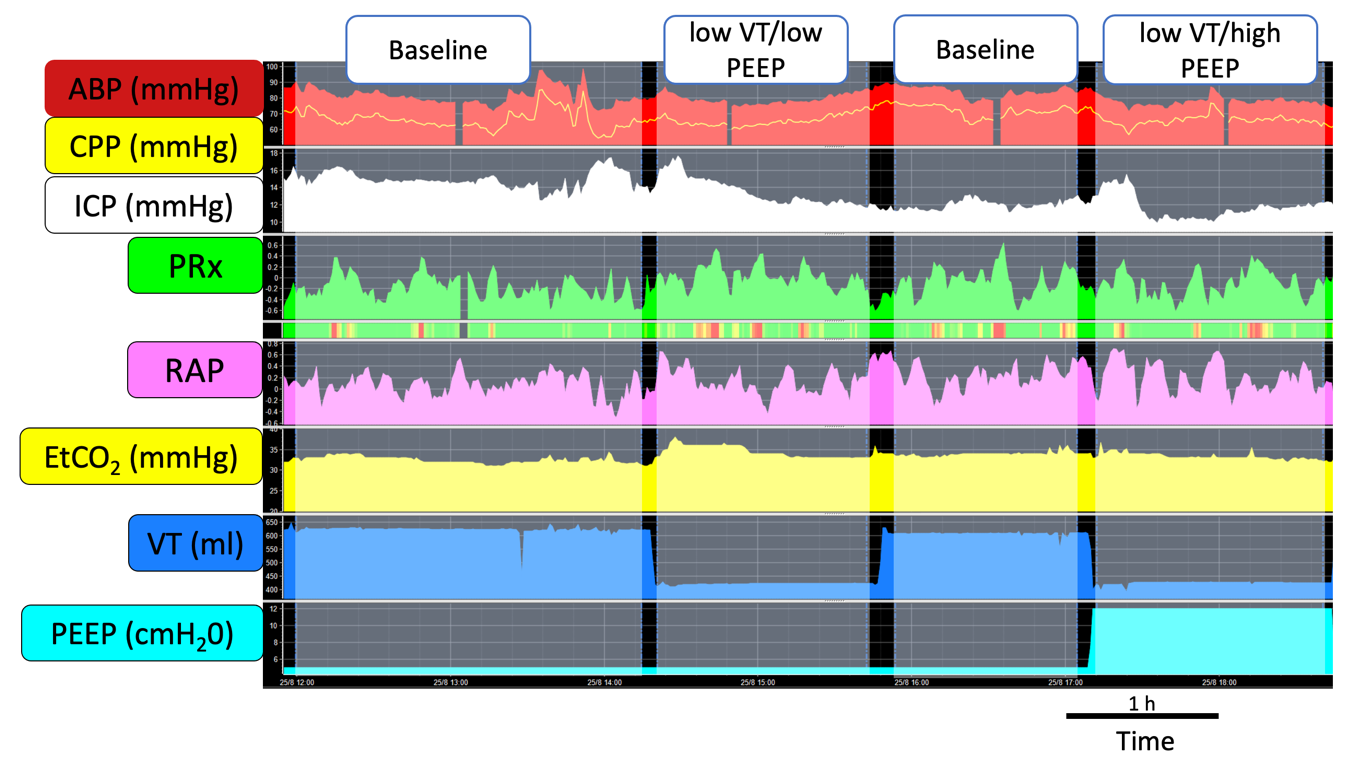
**

B

Insp hold

Exp hold

VT (ml)

Flow (l/s)

Paw (cmH_2_0)

Pes (cmH_2_0)

TPP (cmH_2_0)


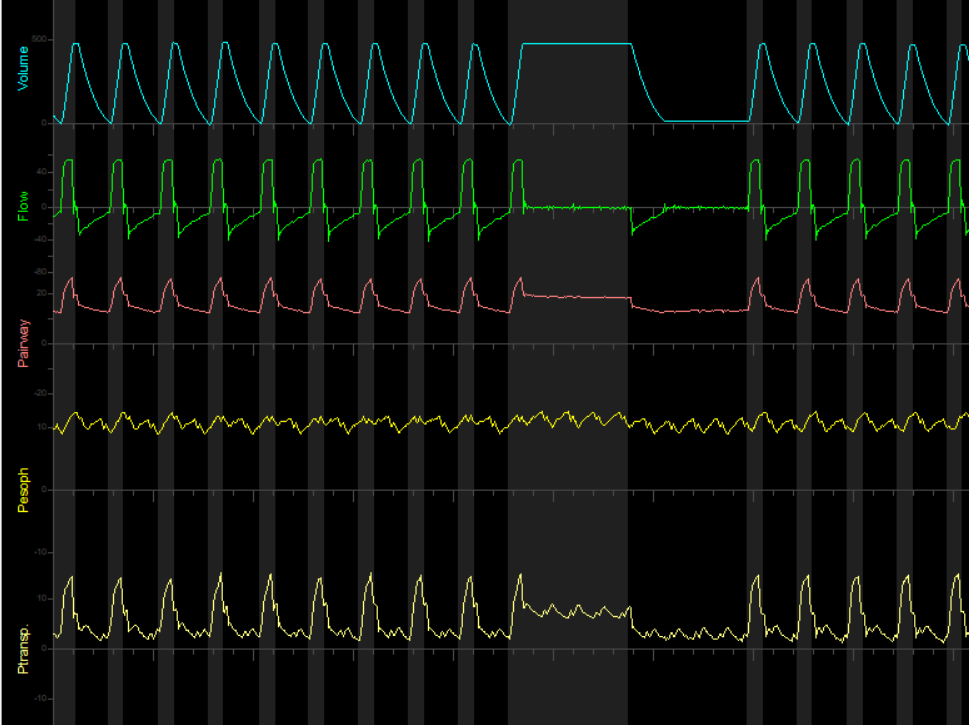


1. **Neuromonitoring screenshot.** Time trends of physiological variables and study periods are represented. ABP, arterial blood pressure; CPP, cerebral perfusion pressure; ICP, intracranial pressure; PRx, Pressure reactivity index; RAP, compensatory reserve index; EtCO_2_, end-tidal CO_2_; VT, tidal volume; PEEP, positive end-expiratory pressure.
2. **Respiratory pressure monitoring screenshot displaying** VT, tidal volume; Paw, airway pressure; Pes, oesophageal pressure; TPP, transpulmonary pressure; Insp, inspiratory; Exp, Expiratory.
3. **Fig. S 2-**


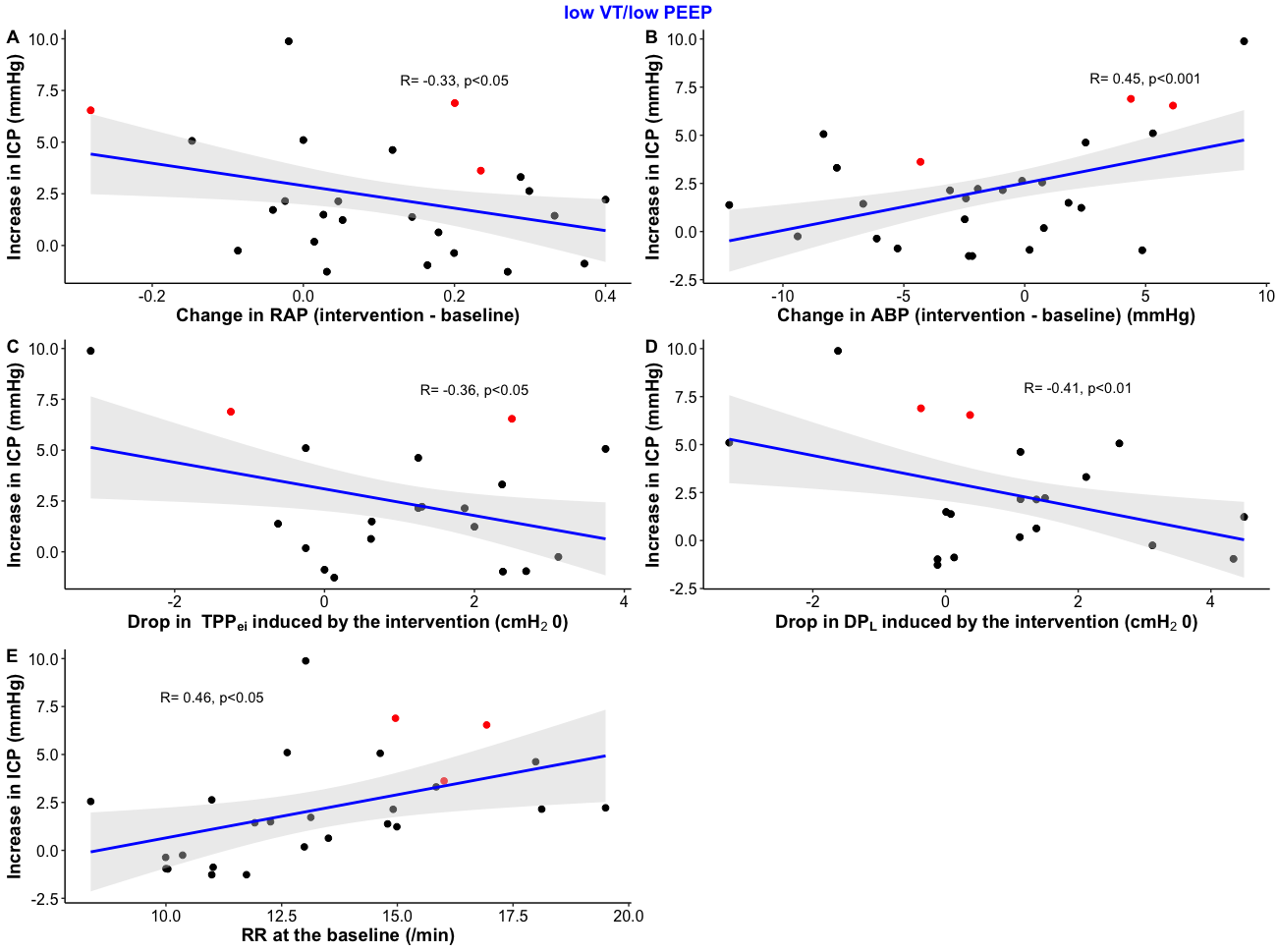


Scatterplots showing the associations between the increase in ICP and changes in RAP (A), ABP (B), TPP_ei_ (C), DP_L_ (D) induced by the intervention low VT/low PEEP, and with RR at the baseline (E).

The increase in ICP correlated negatively with the change in RAP (r = -0.33, p <0.05, ns after Bonferroni correction) and positively with the change in ABP (r = 0.45, p<0.001). The increase in ICP correlated negatively with the decrease in DPL (r = -0.41, p <0.01) and the decrease in TPPei (r = -0.36, p<0.05), and correlated positively with the RR at the baseline (r = 0.45, p<0.05). The change in ICP was also positively associated with the change in ICPsw (r = 0.50, p<0.01) and AMP (r = 0.77, p<0.001), which are not shown in the figure.

ICP, intracranial pressure; RAP, compensatory reserve index; ABP, arterial blood pressure; TPP_ei_ transpulmonary pressure end inspiration, DP_L_, driving pressure of the lung; RR, respiratory rate.

1. **Fig. S 3-**


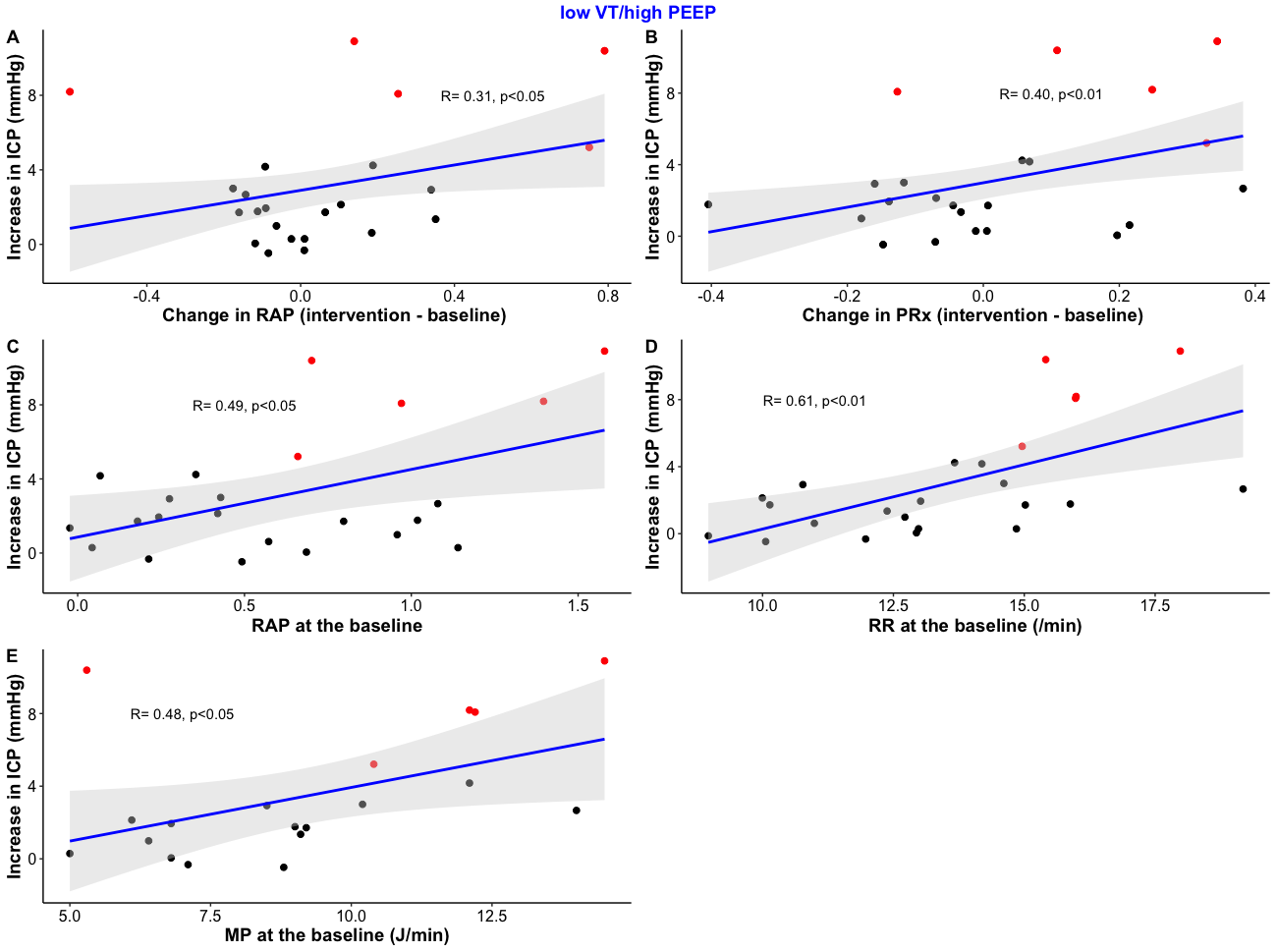


Scatterplots showing the associations between the increase in ICP and changes in RAP (A), PRx (B) induced by the intervention low VT/high PEEP, and with RAP (C), RR (D), MP (E) at the baseline.

The increase in ICP correlated positively with the changes in RAP (r = 0.30, p<0.05, *ns* after Bonferroni correction) and in PRx (r= 0.40, p<0.01, *ns* after Bonferroni correction). The increase in ICP was positively correlated with MP at the baseline (r = 0.48, p<0.05, *ns* after Bonferroni correction) and with RR at the baseline (r=0.61, p<0.01, *ns* after Bonferroni correction). The change in ICP was also associated with the changes in CPP (r = -0.70, p <0.001), ICPsw (r = 0.70, p<0.001) and AMP (r = 0.95, p<0.001), which are not shown in the figure.

ICP, intracranial pressure; RAP, compensatory reserve index; PRx, pressure reactivity index; RR, respiratory rate; MP, mechanical power.

1. **List of abbreviations for the additional material**

ABP: Arterial blood pressure; ABP_sw_ : Slow wave component of ABP; AMP: Amplitude of ICP; C_rs_: Compliance of the respiratory system; C_cw_: Compliance of the chest wall; C_L_: Compliance of the lung; CPP: Cerebral perfusion pressure; CVP: Central venous pressure; DP_rs_: Driving pressure of the respiratory system; DP_cw:_ Driving pressure of the chest wall;

DP_L, abs_: Driving pressure of the lung, absolute value; El_rs_ : Elastance of the respiratory system;EVD: External ventricular drainage; EtCO_2_: End-tidal partial pressure of CO_2_;

FiO_2_:Fraction of inspired oxygen; ICM+: Intensive care monitor +; ICP: Intracranial pressure; ICP_sw_: Slow wave component of ICP; I:E : Inspiratory-to-expiratory time ratio

MP: Mechanical power; PaCO_2_: Arterial carbon dioxide partial pressure; PaO_2_: Arterial oxygen partial pressure; Paw_ei_: End-inspiratory airway pressure; pbw: Predicted body weight;

PEEP: Positive end-expiratory pressure; Pes_ee_: End-expiratory oesophageal pressure; Pes_ee_: End-inspiratory oesophageal pressurepH: Potential hydrogen; PRx: Pressure reactivity index;

RAP: Compensatory reserve index; RR: Respiratory rate; rSO_2_: Regional cerebral oxygen saturation; R_rs_: Resistance of the respiratory system; SpO2: oxygen saturation; TPP_ei_: End-inspiratory transpulmonary pressure, absolute; TPP_ee_: End-expiratory transpulmonary pressure; V_Dalv_: Alveolar dead space; VT: Tidal volume.
